# RLS shows increased resting state sympathetic activity and decreased sympathetic response capability

**DOI:** 10.1007/s00415-025-13285-9

**Published:** 2025-07-30

**Authors:** Christoph Best, Ana Luiza C. Sayegh, Annette Janzen, Wolfgang H. Oertel, Heidrun H. Krämer

**Affiliations:** 1 Department of Neurology, Philipps-University, Marburg, Germany; 2 Department of Neurology, Justus Liebig University, Klinikstrasse 33, 35392 Giessen, Germany

**Keywords:** Restless legs syndrome, Autonomous nervous system, Sympathetic outflow, Microneurography, MSNA

## Abstract

**Background:**

Restless Legs Syndrome (RLS) is a sensorimotor disorder characterized by painful discomfort, an urge to move the legs and circadian and sleep–wake disturbances. The pathophysiology is complex and not fully understood, with inhibitory dysfunction of dopaminergic neurons or increased sympathetic activity being discussed. Aim of this study was to shed light on the association between dysfunction of the autonomic nervous system and RLS.

**Methods:**

6 RLS patients diagnosed according to consensus criteria were compared with 9 gender- and age-matched healthy controls. Patients were clinically characterized using the International RLS Severity Scale (IRLS), the Epworth Sleepiness Scale (ESS) and the RLS Quality of Life Questionnaire (RLSQoL). Multi-unit sympathetic microneurography was performed in the peroneal nerve. Cardiovascular parameters (systolic and diastolic blood pressure, heart rate) and muscle sympathetic nerve activity (MSNA) were assessed at rest and during baroreflex stimulation using lower body negative pressure (LBNP).

**Results:**

MSNA at rest was higher in RLS patients (burst frequency [BF]: 42.4 ± 1.7 bursts/min; burst incidence [BI]: 64.0 ± 2.7 bursts/100 heartbeats) compared to controls (BF: 29.5 ± 1.4 bursts/min, F = 15.332, p = 0.002; BI: 42.9 ± 3.2 bursts/100 heartbeats, F = 21.156, p = 0.001). After baroreflex stimulation, RLS patients had increased absolute values of MSNA (BF: F = 15.096; p = 0.002; BI: F = 21.115; p < 0.001) compared to controls. In RLS patients, BF and BI dropped below baseline values, while in healthy controls BF and BI remained above baseline. After normalization of BF and BI data, the MSNA outflow during baroreflex stimulation was lower in RLS patients than those of healthy controls (BF: F = 4.574; p = 0.002, BI: F = 6.259; p < 0.001).

**Conclusion:**

The current study is the first to directly demonstrate increased sympathetic neuronal activity in RLS. Possible explanations include a dysfunction in inhibitory projections to sympathetic neurons in the intermediate lateral column of the spinal cord or a disease-unspecific effect of sleep deprivation.

## Introduction

Restless legs syndrome (RLS) is a sensorimotor disorder characterized by painful discomfort and a strong urge to move the limbs, especially the legs. It is characterized as a circadian and sleep–wake disorder. The main symptoms are odd sensations and pain in the legs [45]. With a prevalence of about 10% of the population, RLS is one of the most common neurological disorders [42]. RLS is diagnosed according to the consensus criteria of the International Restless Legs Syndrome Study Group (IRLSSG) [3]. As symptoms typically worsen in the evening and during night time, RLS can cause sleep disturbances and thus have a markedly impact on quality of life. In the context of pathophysiological classification, RLS is generally considered to be a network disorder. Alterations in dopaminergic neurotransmission appear to be a possible link between neuroanatomical changes and subsequent symptoms in the patients. A neuropathological study showed a significant reduction of D2 receptors in the putamen region [10]. In addition, genetic factors seem to play a role [44, 52]. Finally, cerebral iron deficiency also plays a crucial role in the pathophysiology [45]. In conclusion, the pathophysiology is complex and yet still not fully understood.

It is speculated that autonomic disturbances might play a part in RLS pathophysiology. To the best of our knowledge, no direct investigation of baroreflex function has been performed in RLS. Using multi-unit sympathetic microneurography, the muscle sympathetic nerve activity (MSNA) that represents a direct neuronal signal can be investigated. The aim of the current study therefore was to directly analyze sympathetic nerve activity under resting condition and to also analyze the response pattern of sympathetic nerve activity after baroreflex stimulation by lower body negative pressure (LBNP).

## Methods

### Study design and participants

In this monocentric prospective and controlled study, 6 RLS patients (three women, age: 54 ± 4 years) and 9 age and gender matched healthy controls (HC) (four women; age: 56 ± 2 years) were enrolled. Patients were recruited from a specialized RLS outpatient clinic of the Department of Neurology at the Philipps-University, Marburg, Germany, after they were diagnosed with RLS. Patients were not allowed to take any medication for their RLS for at least 48 h prior to the microneurography measurement.

Diagnosis of RLS was established on the basis of the consensus criteria of the International Restless Legs Syndrome Study Group (IRLSSG) [3]. Therefore, the following essential criteria had to be fulfilled: (1) Urge to move the legs, usually accompanied or triggered by discomfort or restlessness in the legs. (2) Urge to move the legs and in addition discomfort starting or worsening during rest or inactivity, such as lying down or sitting. (3) Urge to move the legs and in addition discomfort improves partially or even completely with movement such as running, walking or stretching. Improvement has to last as long as the movement continues. (4) Urge to move the legs and additionally discomfort during rest or inactivity has to occur only in the evening or at night time, or it has presented a worsening in the evening or at night time. (5) The occurrence of the above mentioned features cannot be explained by any other medical diagnosis or behavioral condition. In addition the following supportive criteria also were applied: (1) Periodic leg movements during sleep (PLMS) or while awake (PLMW) (on the basis of clinical observation). (2) Improvement of symptoms by medication that contains levodopa or a dopamine agonist. (3) Positive family history of first-degree relatives also suffering from RLS. (4) Absence of impairing daytime sleepiness (as opposed to sleep disorders with a tendency to fall asleep throughout the day).

The RLS patients were compared to 9 HC. Those were recruited through public announcements within the university. The inclusion criteria for HC were: 1) empty history for neurological disorders; 2) unremarkable clinical neurological examination; 3) normal motor nerve conduction studies of the peroneal nerves.

### Exclusion criteria

The following criteria were applied in order to exclude a patient or control person from participating in the study: 1) previous damage to the left peroneal nerve, as proven by electroneurography; 2) current application of oral or parenteral anticoagulation; 3) diagnosis of Parkinson's disease; 4) diagnosis of polyneuropathy; 5) diagnosis of REM sleep behavior disorder (RBD); 6) patients/participants with a BMI > 30; 7) dopaminergic or opioidergic medication had to be paused at least 48 h before the study, any other medication interfering with the autonomic nervous system counted for exclusion.

### Characteristic of RLS patients

An extensive clinical evaluation was carried out to ensure comparability with other studies of RLS patients. For the clinical assessment of RLS patients the duration of RLS and average daily sleep time evaluation were recorded. Furthermore, the International RLS Severity Scale (IRLS), the Epworth Sleepiness Scale (ESS) and the RLS Quality of Life Questionnaire (RLSQoL) were performed. A detailed description of those routine assessment tools is given elsewhere [1, 26, 38, 48, 49].

### Microneurography and lower body negative pressure (LBNP)

The microneurographic examination was carried out at rest and during LBNP stimulation [39]. Participants were placed in a resting position in the bottom shell of a custom-made lower body LBNP chamber. All examinations were conducted on the left peroneal nerve. The exact localization of the peroneal nerve was defined applying short, weak electrical impulses. The microneurographic needle was inserted through the skin and placed into the sympathetic muscle fascicle of the peroneal nerve and pushed forward until spontaneous sympathetic activity occurred. As reference a second electrode was inserted subcutaneously [46]. Thus, the present study recorded postganglionic multi-unit MSNA directly from the peroneal nerve using microneurography (0.2 mm wire with 5 µm tip). The signals were amplified (× 100,000) and band-pass filtered (700–2000 Hz), followed by a rectification and integration of nerve activity (time constant 0.1 s) to obtain a mean voltage reading [15]. After MSNA baseline measurement for a period of an interval of 15 min, LBNP stimulation was activated.

To activate the LBNP induced baroreflex stimulation, the LBNP chamber was hermetically sealed. A continuously adjustable industrial suction device (Bosch, Germany) was connected to the hermetically sealed chamber. The extent of the negative pressure stimulation was controlled by continuous measurement using a pressure manometer. Any necessary corrections were made using the continuously adjustable setting of the suction device. A target negative pressure of − 40 mmHg was used. The method has been established and standardized [2, 39].

In addition the heart rate (HR) was monitored by a continuously electrocardiogram. Furthermore, systolic and diastolic blood pressure (SBP, DBP) was evaluated every minute by a digital sphygmomanometer (WEPA, Hillscheid, Germany).

### Data analysis

The aim parameters for further statistical analyses were derived from the MSNA recordings, the HR and BP values. MSNA results were recorded as raw data and then analyzed offline by a homebuilt computer-controlled system [29]. MSNA bursts were identified automatically and individually confirmed by visual control. Two parameters were then calculated: (a) Burst frequency (BF, bursts per minute) of muscle sympathetic bursts as measure of sympathetic activity processed to the effector organs was calculated and (b) burst incidence (BI, bursts per 100 heart beats) as a measure of central sympathetic drive was calculated. In addition to the analyses of absolute values, a normalization with respect to the pre-stimulation resting period was calculated. Thereby presenting the relative differences in MSNA activity between rest and baroreflex stimulation by LBNP.

### Statistical analyses

Testing for normal data distribution and homogeneity of the sample, in a first step Kolmogorov–Smirnov and the Levene’s tests were calculated. Testing for differences between RLS patients and HC a single factor variance analysis (ANOVA) was conducted. Greenhouse–Geisser correction was used to correct for violation of sphericity. All statistical analyses were performed using SPSS 29 (IBM, CA, USA). Data are shown as mean ± SD. F-values indicate the effect strength of group differences, p < 0.05 indicated significance of group differences. Given the small group size, a post-hoc power analysis was conducted based on the observed effect sizes in order to contextualize the strength of the results. The software G*Power (version 3.1.9.7, Heinrich Heine University, Duesseldorf, Germany; [18]) was used for this purpose.

## Results

### Basic and clinical characteristics

There were no differences between the groups in terms of age, gender, or baseline clinical outcomes. Detailed data are shown in Table 1.

**Table 1:** Basic and clinical data

Variables and clinical data	RLS (n = 6)	Control (n = 9)	Statistics
Age, yrs, mean	54 ± 4	56 ± 2	n.s., p = 0.57
Female patients (%)	3 (50)	4 (44.4)	n.s., p = 0.70
Weight [kg]	79.5 ± 5.2	79.2 ± 2.6	n.s., p = 0.96
Height [m]	1.73 ± 0.02	1.72 ± 0.03	n.s., p = 0.96
BMI [kg/m^2^]	26.7 ± 1.8	26.7 ± 0.7	n.s., p = 0.98
HR [beats/min]	65 ± 2	70 ± 4	n.s., p = 0.33
SBP [mmHg]	133 ± 7	137 ± 2	n.s., p = 0.51
DBP [mmHg]	82 ± 5	86 ± 1	n.s., p = 0.29
MBP [mmHg]	99 ± 5	103 ± 1	n.s., p = 0.35
Baseline MSNA			
Rest MSNA BF [burst/min]	42.4 ± 1.7	29.5 ± 1.4	F = 15.332, p = 0.002
Rest MSNA BI [burst/100 heart beats]	64.0 ± 2.7	42.9 ± 3.2	F = 21.156, p = 0.001

No significant differences could be revealed between the RLS and the control group concerning clinical data, heart rate and blood pressure

Significant differences were found for MSNA outflow by burst frequency (BF) and burst incidence at baseline measurement

Values are mean ± SD

*RLS* Patients with restless legs syndrome, *M* Male, *F* Female, *kg* Kilograms, *BMI* Body mass index, *HR* Heart rate, *SBP* Systolic blood pressure, *DBP* Diastolic blood pressure, *MBP* Mean blood pressure, *MSNA* Muscle sympathetic nerve activity, *BF* Burst frequency, *BI* Burst incidence

The mean duration of RLS in the patients was 15 years (± 2 years), and the mean sleep time per night was 6 h (± 1 h). All patients were taking dopaminergic medication, opioid medication or a combination of both. Details of these specific characteristics are given in Table 2.

**Table 2:** Patient characteristics

Patient	Gender, age	Duration RLS (y)	Sleep duration (h)	IRLS	ESS	RLSQoL	Medication
1	F, 45	15	7	19	10	48	Pramipexole, Tramadol
2	M, 51	17	6	32	23	36	Levodopa
3	M, 64	20	7	26	12	54	Rotigotine
4	F, 68	20	5	7	3	80	Rotigotine, Levodopa
5	M, 49	10	6,5	27	7	32	Tilidine
6	F, 45	8	2,5	35	23	26	Levodopa

Characteristics of the included RLS patients are presented

*IRLS* International RLS Severity Scale, *ESS* Epworth sleepiness scale, *RLSQoL* Restless legs syndrome quality of life questionnaire, *y* years, *h* hours

The IRLS showed a pathological group mean of 24 (± 5), comprising severe RLS-associated symptoms. Two patients reported very severe symptoms (IRLS score: 32, 35), two patients severe symptoms (IRLS score: 26, 27), one patient moderate symptoms (IRLS score: 19) and one patient only mild symptoms (IRLS score: 7) (Table 2).

ESS showed an elevated group mean of 13 (± 4). Two patients had excessive daytime sleepiness (ESS score: 23, 23), one patient mild daytime sleepiness (ESS score: 12), three patients did not have increased daytime sleepiness (ESS scores: 3, 7, 10) (Table 2).

Assessing the RLSQoL, three patients reported severe impairment (RLSQoL scores: 36, 32, 26), two patients a moderate impairment (RLSQoL scores: 54, 48) and one patient mild symptoms (RLSQoL score: 80) (Table 2).

### Detailed clinical characteristics, comorbidities and medication

Table 3 provides a detailed breakdown of all comorbidities and additional medications taken alongside RLS medication. As the study protocol investigated sympathetic neuronal activity, we identified possible confounders of sympathetic activity such as comorbidities and categorized them as follows: a) cardiac and autonomic comorbidities, b) psychiatric and psychological comorbidities and c) other comorbidities (See Table 3 for details).

**Table 3:** Detailed clinical characteristics, comorbidities and medication

Patient	Cardiac conditions/autonomic disturbances	Psychiatric/psychological comorbidities	Other comorbidities	None-RLS medication
1	none	none	-Hypothyroidism -Chronic lower back pain -Osteoarthritis of the knee joint -Migraine	-Levothyroxine
2	Former atrial fibrillation	none	-Ulnar neuropathy -Carpal tunnel syndrome - Iron deficiency	-Vitamin D3
3	none	none	-Type 2 diabetes mellitus -Chronic lower back pain -Hypercholesterolemia	-Rosuvastatin -Sitagliptin and metformin
4	Arterial hypertension	none	none	none
5	none	Mild depressive episode	-Obstructive sleep apnea -Bronchial asthma -Tinnitus -degenerative cervical spine disease	none
6	none	none	-Former cervical disc herniation -non-celiac gluten sensitivity	none
HC	Cardiac conditions/autonomic disturbances	Psychiatric/psychological comorbidities	Other comorbidities	None-RLS medication
1	Arterial hypertension	none	-Hypercholesterolemia	-Ramipril -Simvastatin
2	none	none	-Migraine	None
3	none	none	-Chronic lower back pain	None
4	none	none	-Hypothyroidism	-Levothyroxine
5	none	none	none	none
6	none	Former mild depressive episode	none	none
7	Arterial hypertension	none	-Type 2 diabetes mellitus -Hypercholesterolemia	-Valsartan -Metformin -Simvastatin
8	none	none	-Hip arthrosis	none
9	none	none	none	none

Table 3 presents a detailed description of all comorbidities and the medications taken in addition to RLS medication

Comorbidities were categorized into a) cardiac and autonomic comorbidities, b) psychiatric and psychological comorbidities, and c) other comorbidities

### Muscle sympathetic nerve activity during baseline measurements

MSNA at baseline showed significant differences between patients and HC: Both BF (RLS: 42.4 ± 1.7 vs. HC: 29.5 ± 1.4; F = 15.332; p = 0.002) and BI were significantly increased in the patient group (RLS: 64.0 ± 2.7 vs. HC: 42.9 ± 3.2; F = 21.156; p = 0.001) (Table 1).

### Muscle sympathetic nerve activity during baroreflex stimulation

#### MSNA burst frequency (BF)

**Fig. 1:**
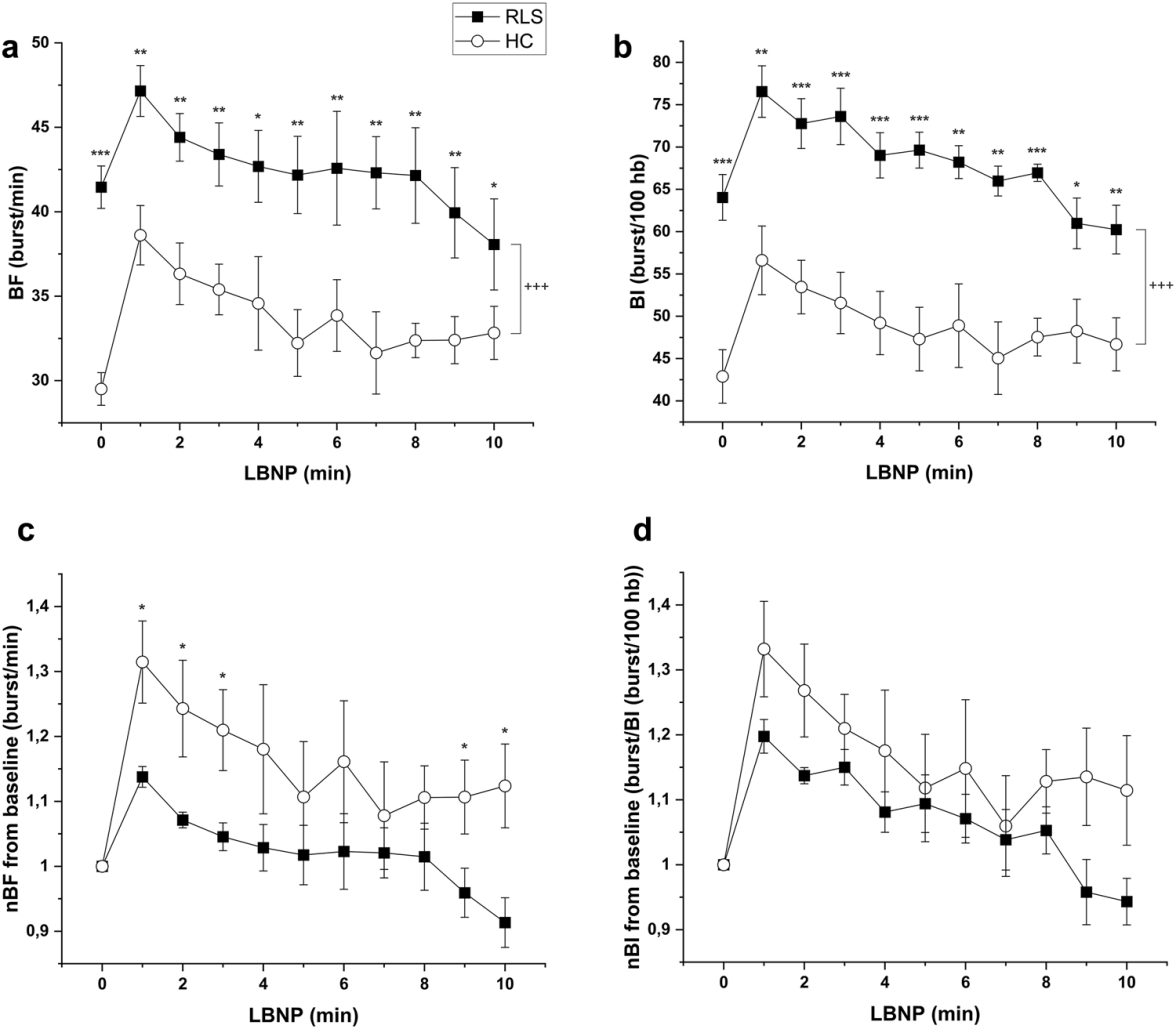
Muscle sympathetic nerve activity by BF (1.a) and BI (1.b) at rest (minute 0) and following baroreflex stimulation (LBNP) at minutes 1–10. In addition, changes in MSNA compared to baseline (resting period) are presented after offset elimination at each time point during baroreflex stimulation (LBNP) by BF (1.c) and in BI (1.d) in the RLS patients compared to healthy controls. BF = burst frequency; BI = burst incidence; LBNP: lower-body negative pressure; RLS = Restless Legs disorder. ANOVA: + = p < 0.05; + + = p < 0.01; + + + = p < 0.001; T-Test vs. healthy control: * = p < 0.05; ** = p < 0.01; *** = p < 0.001

During baroreflex stimulation, time course analysis revealed a significantly higher BF in RLS compared to HC (ANOVA, F = 15.096; p = 0.002). Post hoc tests revealed increased BF outflow in the patients at every single time point during stimulation (Fig. 1a). The course of reactive BF after the LBNP onset differed significantly between the groups, showing two types of response patterns (F = 5.332; p < 0.001). While BF of RLS patients started with a significantly higher baseline (initial BF = 42.4 ± 1.7), it dropped below the baseline at the end of the stimulation (final BF = 38.1 ± 2.7). In contrast, the BF of HC started with a significantly lower baseline (initial BF = 29.5 ± 1.4) and stayed above baseline (final BF = 32.8 ± 1.6) (Fig. 1a).

In a second step of the analysis, the MSNA results were normalized to baseline in order to reveal the relative change induced by baroreflex stimulation. There was no significant difference between the two groups in the absolute values of normalized BF (ANOVA: F = 3.120; p = 0.101). Interestingly, the relative increase compared to baseline was higher in the HC group compared to RLS throughout the stimulation period (ANOVA: F = 4.574; p = 0.002). However, post-hoc t-test calculations showed a significant difference at minute 1, 2, 3, 9 and 10 (Fig. 1c). While BF in RLS patients decreased below baseline at the end of stimulation, BF in HC stayed above baseline, even after normalization of the data (Fig. 1c).

#### MSNA burst incidence (BI)

A similar pattern of MSNA during baroreflex stimulation was seen for sympathetic outflow as characterized by BI. RLS patients had significantly higher central sympathetic drive than HC (BI: F = 21.115; p < 0.001). BI showed a significant increase in RLS patients at every time point throughout the LBNP stimulation (Fig. 1b). The course of the reactive BI also showed a significantly different response pattern between groups (F = 8.401; p < 0.001). In RLS patients, BI dropped below baseline at the end of stimulation (initial BI: 64.0 ± 2.7 vs. final BI: 60.2 ± 2.9), whereas in HC central sympathetic drive as measured by BI stayed above baseline (initial BI: 42.9 ± 3.2 vs. final BI: 46.7 ± 3.1) (Fig. 1b).

After normalization the relative increase did not differ significantly between groups in the absolute values of normalized BI (ANOVA: F = 1.384; p = 0.261). The relative increase in comparison to baseline again was higher in HC compared to RLS as evoked by LBNP stimulation (ANOVA: F = 6.259; p < 0.001) (Fig. 1d). Post hoc analysis revealed a trend within the last 2 min of stimulation but failed significance level. While the BI in RLS patients decreased below baseline, BI in HC stayed above baseline (Fig. 1d).

#### Post-hoc power analyses

Raw data analyses revealed a test power of 82.8% for burst frequency (BF; effect size d: 2.01; test power: 82.8%) and a test power of 93.4% for burst incidence (BI; effect size d: 2.50; test power: 93.4%), underlining the strength of the results. Normalized data analyses after offset elimination revealed, that sample size was too small, to discover stronger significant effects: test power of 12.4% for normalized burst frequency (nBF; effect size d: 0.2; test power: 12.4%) and a test power of 13.2% for normalized burst incidence (nBI; effect size d: 0.22; test power: 13.2%).

### Analyses of blood pressure and heart rate

When HR and BP were analyzed at baseline no difference between the groups was observed (see Table 1). After start of LBNP stimulation, no difference between the groups could be detected over the entire stimulation period (HR: F = 0.918; p = 0.355; SBP: F = 0.473; p = 0.504; DBP: F = 1.130; p = 0.307). Furthermore, no significant changes in HR or BP could be detected within the two groups during the stimulation period (HR: F = 0.619; p = 0.641; SBP: F = 0.789; p = 0.534; DBP: F = 0.488; p = 0.769).

## Discussion

To the best of our knowledge, this is the first study to demonstrate direct measurement of sympathetic neuron activity by microneurography under rest and by LBNP baroreflex stimulation. RLS patients showed significantly increased MSNA at rest and during stimulation compared to HC. However, the relative increase of sympathetic outflow was lower in RLS compared to HC. Furthermore, the pattern of sympathetic reactivity to LBNP stimulation differed. The other monitored cardiovascular parameters did not differ since those parameters underlie multiple regulatory mechanisms and do not represent a pure sympathetic neuronal signal such as MSNA. As autonomic dysfunction has been implicated in the pathophysiology of RLS, alterations in the baroreflex circuit and especially in elevated resting state sympathetic activity, may at least play a partial role within the proposed network underlying RLS.

### Autonomic dysfunction in RLS

Significant autonomic disturbances in RLS were presented by a number of studies: Rocchi's research showed no change in cardiovascular response by pramipexole treatment, suggesting a non-dopaminergic mechanism of autonomic dysfunction in RLS [37]. Izzi et al. found increased blood pressure and lower Valsalva ratio in RLS. They suggested this could increase cardiovascular risk in RLS [23]. Isak et al. compared RLS patients and HC using nerve conduction studies, sympathetic skin response and heart rate variability, revealing no differences [22]. Thus suggesting that autonomic complaints in RLS may be a result of a dysfunction of the dopaminergic caudal diencephalic A11 group [22]. Schulte et al. reported an association between increased sympathetic activity and RLS [40]. Walters and Rye also described a relationship between increased sympathetic activity and RLS. They focused on inadequate dopaminergic A11 diencephalic inhibition of preganglionic sympathetic neurons [50]. Chenini and coworkers studied multiple autonomic symptoms in a large cohort of RLS patients and concluded a global autonomic dysfunction [8]. Jin and colleagues confirmed the association between increased sympathetic activity and comorbid cardiovascular risk [25]. Erdal et al. demonstrated clear autonomic dysfunction in RLS patients using sympathetic skin response and RR interval variability [17].

Overall, the discussion focuses on inhibitory dysfunction of the dopaminergic diencephalic A11 group, increased sympathetic activity caused by RLS and impaired sensorimotor integration.

### Neuro-anatomical considerations

Our results may be explained by inhibitory dysfunction of the dopaminergic diencephalic A11 group resulting in dysfunction of the baroreflex as part of the RLS pathophysiology. Research in animals has studied the neuroanatomical basis and connections controlling sympathovagal and baroreflex tone [12, 13]. The NTS is the main relay station in the baroreflex circuit, projecting to other regions, especially the RVLM. The activity of sympathetic neurons is controlled via excitatory supraspinal descending projections, originating in defined brainstem and hypothalamic regions: RVLM, rostral ventromedial medulla, midline medulla, A5 cell group of the pons, and paraventricular nucleus of the hypothalamus (PVN). In this network, the RVLM and PVN play primary roles, with the PVN acting indirectly via RVLM intercalated projections [41].

The RVLM is controlled by neurons that release glutamate and GABA. Glutamate neurons originate from the pontine reticular formation [30], the pontine lateral tegmental area and the PVN of the hypothalamus [4]. GABA neurons originate from the baroreceptors, the caudal ventrolateral medulla and the caudal pressor area (CPA).

The diencephalic A11 region may be involved in the origin of RLS symptoms, and the dopaminergic innervation of the spinal cord originates mainly from this area [9, 36]. Projections from the A11 region contribute to the control of locomotion [19] and pain control [34], and the neurons send projections to various brain regions, including the dorsal raphe [27, 35]. Both the direct projections to the spinal cord and the projections to the dorsal raphe may link the pathophysiology of RLS and the observed increased sympathetic outflow. One generator of periodic limb movements in RLS patients can be found in the brainstem area of the reticular formation, as shown in an early functional MRI study by Bucher and colleagues [5], displaying a further possible neuro-anatomical linkage between RLS and sympathetic excitation. In conclusion, a dysfunctional A11 group could disinhibit the reticular formation neurons, which may increase excitatory RVLM projection of preganglionic intermediate lateral column neurons. Alternatively, excitatory pontine reticular formation projections could increase RVLM activity directly, but further investigations are needed to understand the exact neuro-anatomical interaction between RLS and increased sympathetic outflow.

Another key point: while sympathetic outflow is increased in RLS, any further increase shows a ceiling effect, as the relative MSNA increase in RLS is lower than in HC, suggesting the baroreflex plays a role in maintaining body homeostasis and pointing to a functional change rather than a structural one in RLS. However, further research is needed as the exact mechanisms are still unclear.

### Sleep disturbances underlying sympathetic excitation

Another possible explanation of our results may be that sleep disturbances and inadequate sleep quality are associated with autonomic dysfunction. Short sleep duration has been associated with an increase in cardiovascular risk, with autonomic nervous system dysfunction being an important contributor. Sleep disorders are characterized by sympathetic excitation with sympathetic and baroreflex dysfunction [21].

There are contradictory correlations between sleep disorders and sympathetic activation. A significant increase in sympathetic outflow was demonstrated for obstructive sleep apnea and iRBD [6, 20, 31, 32, 39]. No effect in MSNA at resting condition compared to HC was found in insomnia; however, MSNA increase was demonstrated after autonomic stimulation [7, 15]. A decrease in sympathetic outflow by reduced MSNA was found in narcolepsy [14, 16, 24]. Similarly, decreased sympathetic outflow was observed after sleep deprivation in HC [28], 33. A relationship between sleep disturbance or impaired sleep duration/quality and altered autonomic nervous system activity thus is evident. However, no unidirectional response to reduced sleep duration or poor sleep quality exists. Sleep disturbances in RLS therefore cannot be the sole factor explaining sympathetic overexcitation in RLS patients.

### Effects of opioidergic and dopaminergic medication

The increased MSNA in RLS could be explained by dopaminergic or opioidergic medication, but the data is limited. Watso and coworkers showed that morphine had no effect on MSNA during a cold pressor test, while Cook et al. showed that opioidergic medication had no effect on MSNA. Takeuchi and colleagues found that levodopa increased MSNA during a HUTT [11, 43, 51]. In our study, the patients'medications are likely to have had no influence on the results since they had not taken dopaminergic or opioidergic medication for at least 48 h.

### Limitations

However, our study has some limitations. One limitation is the small number of patients recruited. Given the small sample size as potential confounder of results, a post-hoc power analysis was conducted. Hereby we could demonstrate, that raw data analyses depicted a high result strength. For an analysis of the normalized data, the sample was too small to detect further significant results based on the given effect size, which meant that further target effects could not be uncovered.

In addition, we were able to demonstrate increased MSNA in every single of the RLS patients and MSNA is a very stable parameter and therefore provides reliable data even in a small cohort [46, 47].

In addition, sympathetic microneurography of the peroneal nerve is laborious and time-consuming, it is operator-dependent and not well tolerated by patients. Consequently, it is unlikely to become a widely used measure of autonomic function.

Furthermore, anxiety was not evaluated in the present study. Anxiety frequently co-occurs with RLS and is capable of increasing sympathetic outflow. Therefore, the potential influence of anxiety on MSNA was not analyzed.

Furthermore, patients were required to discontinue their opioid or dopaminergic medication for 48 h. This could induce withdrawal symptoms, exacerbate RLS and consequently increase anxiety levels, potentially interfering directly with autonomic function. Withdrawal could therefore affect the results of sympathetic outflow. Given the short elimination half-lives of the medications used by the patients in the current study, the potential for withdrawal effects after 48 h cannot be ruled out. In contrast, additional cardiovascular parameters such as heart rate (HR) and blood pressure (BP) did not differ from those of healthy controls (HC). In manifest withdrawal, an increase in these parameters would have been evident, making withdrawal a rather unlikely confounding factor. To exclude the effects of long-term medication or withdrawal, RLS patients should be examined at diagnosis and before the initiation of medication.

## Conclusion

Compared to healthy controls, patients with restless legs syndrome (RLS) exhibit significantly increased peripheral postganglionic sympathetic activity. Following autonomic stimulation using LBNP, this activity can be elevated further for a short time, but falls below baseline levels by the end of the stimulation period. The relative extent to which the sympathetic nervous system responds to LBNP stimulation is significantly lower than that observed in healthy controls. In summary, although the resting sympathetic activity level is elevated and quickly reaches a ceiling effect, this ultimately does not lead to haemodynamic relevance, as heart rate (HR) and blood pressure (BP) can still be compensated for by other mechanisms. The exact underlying pathophysiological mechanism remains to be elucidated. One possible explanation is impaired inhibitory projection of dopaminergic A11 neurons to preganglionic RVLM neurons, which project to the intermediate collateral column of the spinal cord. This high pitched resting state sympathetic activity may represent a correlate of the low-threshold unpleasant perceived sensorimotor integration in RLS patients.

## Abbreviations

ANOVA: Analysis of variance
BI: Burst incidence
BF: Burst frequency
BMI: Body mass index
BP: Blood pressure
DBP: Diastolic blood pressure
ESS: Epworth sleepiness scale
HB: Heart beats
HC: Healthy controls
HR: Heart rate
HRV: Heart rate variability
IRLSSG: International restless legs syndrome study group
IRLS: International RLS severity scale
LBNP: Lower body negative pressure
MBP: Mean blood pressure
MSNA: Muscle sympathetic nerve activity
NTS: Nucleus tractus solitarius
PLMS: Periodic leg movements during sleep
PLMW: Periodic leg movements while awake
PVN: Periventricular nucleus of hypothalamus
RBD: REM sleep behaviour disorder
RLS: Restless legs syndrome
RLSQoL: Restless legs syndrome quality of life questionnaire
RVLM: Rostral ventrolateral medulla
SBP: Systolic blood pressure
